# Refractory severe aplastic anemia in a child: treatment failure and access barriers in a low-resource setting

**DOI:** 10.1097/MS9.0000000000004007

**Published:** 2025-10-07

**Authors:** Zeinab Karaki, Chrystelle Chemaly, Jana Kotaich, Ali Shmeis, Raghida Dhayni

**Affiliations:** a Faculty of Medical Sciences, Lebanese University, Hadath, Lebanon; b MEDICA Research Investigation, Hadath, Lebanon; c Faculty of Medical Sciences, Department of Pediatrics, Beirut Arab University, Lebanon; d Pediatric Hematology-Oncology Unit, Hammoud Hospital University Medical Center (HHUMC), Saida, Lebanon; e Hammoud Hospital University Medical Center (HHUMC), Saida, Lebanon

**Keywords:** aplastic anemia, bone marrow failure, case report, hematopoietic stem cell transplant, immunosuppressive therapy, pediatric

## Abstract

**Introduction and importance:**

Aplastic anemia (AA) is a rare but potentially fatal hematologic disorder in children, marked by bone marrow failure and pancytopenia. Early diagnosis and access to curative treatment such as hematopoietic stem cell transplantation (HSCT) are crucial. In low-resource settings, limited access to HSCT poses significant challenges.

**Case presentation:**

We present a 7-year-old girl with spontaneous ecchymoses and purpura following an upper respiratory infection. Laboratory tests revealed severe pancytopenia, and bone marrow biopsy showed <5% cellularity, consistent with severe AA. Infectious, autoimmune, and malignant causes were excluded. She received immunosuppressive therapy with rabbit anti-thymocyte globulin, cyclosporine A, and corticosteroids but failed to show hematologic recovery. HSCT was not an option due to financial constraints, and she remained transfusion-dependent.

**Clinical discussion:**

AA is often immune-mediated, and IST is a standard option when HSCT is unavailable. However, responses vary, particularly in pediatric cases. This case illustrates the limited effectiveness of IST in some patients and highlights how socioeconomic barriers hinder access to definitive treatment, impacting outcomes.

**Conclusion:**

This case underscores the urgent need for affordable curative therapies like HSCT in low-income settings. It also emphasizes the variable response to IST and the role of systemic inequities in limiting care for children with life-threatening hematologic diseases.

## Introduction

Aplastic anemia (AA) is a life-threatening hematologic disorder characterized by peripheral pancytopenia and hypocellularity of the bone marrow^[1]^. The yearly incidence of AA is estimated at around two cases per million in Europe and North America, increasing by a factor of 2–3 in East Asia^[2]^. While it is rare and can affect individuals of any age, its diagnosis in the pediatric population is concerning due to its potential fatal complications and rapid progression if not diagnosed early and treated effectively^[1]^. The pathogenesis of AA is most commonly due to autoimmune mechanisms that target the progenitor stem cells, leading to pancytopenia^[3]^. It can also be triggered by environmental toxins, viral infections, or medications^[1,3]^. Typical treatment includes hematopoietic stem cell transplantation (HSCT); however, in the case of an unavailable matched donor, immunosuppressive therapy (IST), combining antithymocyte globulin (ATG) with cyclosporine and corticosteroids is used^[4]^. This treatment approach has shown the best results in pediatric patients, with many responding favorably within weeks to months. However, cases of refractory IST can exist and be challenging to treat, especially in low-resource settings where access to HSCT is limited.

**HIGHLIGHTS**

- Reports a rare case of severe aplastic anemia in a 7-year-old child.
- Failure of standard triple immunosuppressive therapy in a low-resource setting.
- Diagnosis confirmed by bone marrow biopsy showing <5% cellularity.
- Financial constraints limited access to curative hematopoietic stem cell transplant.
- Underscores the need for affordable and accessible therapies in resource-limited countries.

Here, we present a unique and complex case of a 7-year-old girl who developed severe AA following a presumed viral illness and failed to respond to standard triple IST, highlighting the diagnostic complexities, treatment limitations, and the urgent need for accessible curative options in resource-constrained environments.

This case report has been reported in line with the TITAN Guideline Checklist 2025^[5]^.

## Case presentation

A 7-year-old female, previously healthy, presented to the hospital with diffuse ecchymosis and purpura on her lower extremities, which had been present for a few days. Her medical history revealed an upper respiratory tract infection (URTI) 2 weeks prior, which had resolved spontaneously. The patient’s family and surgical histories were unremarkable, and she denied any previous episodes or a family history of bleeding disorders.

Upon admission, her vital signs were stable. Physical examination revealed visible ecchymosis on the lower extremities and purpura on the neck. There were no palpable lymph nodes, hepatomegaly, or splenomegaly.

Initial laboratory results showed significant thrombocytopenia (8 × 10^3^/μL), a low red blood cell (RBC) count (3.41 × 10^6^/μL), and anemia (hemoglobin 9.5 g/dL) (Table 1). The chemistry panel, liver function tests, renal function tests, coagulation panel, and other electrolyte results were all normal. The peripheral blood smear demonstrated slight anisocytosis, with a tendency towards microcytic hypochromic RBCs, cigar-shaped cells, target cells, and a few fragmented RBCs.

**Table 1:** Changes in hematologic parameters on admission and three months after treatment

	On admission	Three months later	Normal range
WBCs	3.81	2.66	(4–11)/mm^3^
ANC	571.5	198	(1500–8000) cells/mm^3^
RBCs	3.41	2.84	(4.34–5.6) million/mm^3^
Hb	9	7.8	(13.5–17.5) g/dL
Hct	23.6	20.9	(38.6–49.2)%
MCV	76.1	74.6	(80–100) µm^3^
Platelets	8	5	(150–450)/mm^3^

ANC, absolute neutrophilic count; CBC, complete blood count; Hbg, hemoglobin; Hct, hematocrit; MCH, mean cell hemoglobin; MCV, mean cell volume; RBCs, red blood cells; WBCs, white blood cells.

A direct and indirect Coombs test yielded negative results, ruling out autoimmune hemolytic anemia (AIHA) and immune thrombocytopenic purpura (ITP), and the absence of schizocytes in the blood smear, along with the absence of neurological defects and renal impairment, ruled out thrombotic thrombocytopenic purpura (TTP). Additionally, vitamin B12 was within normal limits, ruling it out as a possible cause of this pancytopenia.

The patient was initially treated with high-dose Solumedrol for 2 days, but there was no improvement in the platelet count. IVIG was then administered, and the platelet count remained unchanged after 48 hours. These findings reinforced the exclusion of AIHA or ITP as the possible diagnosis. Gammaglobulin electrophoresis revealed normal values (IgM 1.5 g/L, IgG 10.9 g/L, IgA 1.2 g/L) precluding monoclonal gammopathies or plasma cell dyscrasias.

The reticulocyte count was 0.58% after correction for hematocrit, and lactate dehydrogenase levels were within normal limits, suggesting hypoproliferation of the bone marrow rather than hemolysis.

**Figure 1.**
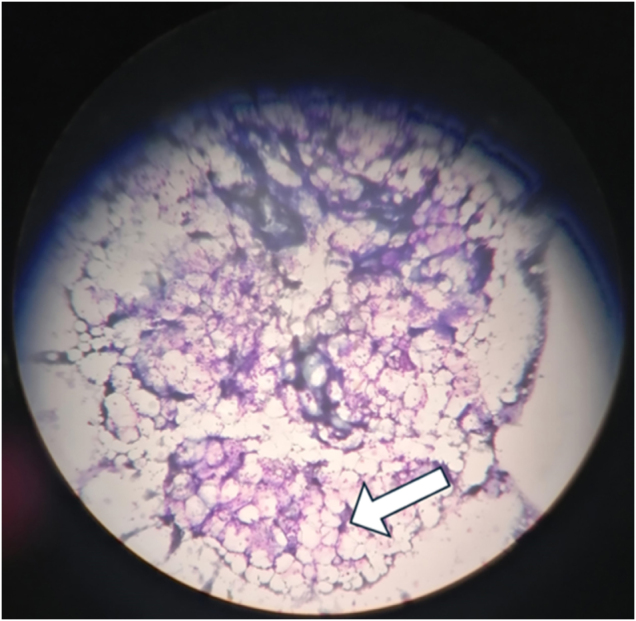
Bone marrow aspiration is particulate and markedly hypocellular, consistent with aplastic anemia. No abnormal cells were seen. Arrow pointing toward an area of hypocellularity.

A bone marrow aspirate was performed, revealing findings consistent with AA. The report noted few hypocellular spicules, absent megakaryocytes, a depressed erythroid series, decreased granulocytes, rare blasts, and predominantly mature lymphocytes (Fig. 1). Bone marrow biopsy confirmed the suspected diagnosis – AA - where less than 5% cellular bone marrow with panhypoplasia was reported.

Further test results (Table 2) were negative for any infections, except that the HSV IgM type 2 level is borderline. However, she was positive for the herpes virus (types 1 and 2) and parvovirus IgG antibodies, which were likely from a previously cleared infection.

**Table 2:** Infectious serology profile for aplastic anemia workup

Test	Result/interpretation
HBsAg	Non-reactive
HCV antibody	Non-reactive
HIV 1,2	Non-reactive
HSV type 1 IgM	Negative
HSV type 1 IgG	Positive
HSV type 2 IgM	Borderline
HSV type 2 IgG	Positive
EBV IgM	Negative
Parvovirus B19 IgG	Positive
Parvovirus B19 IgM	Negative
CMV IgM	Negative

The patient was not able to undergo Allogeneic Hematopoietic Stem Cell Transplant (HSCT) due to financial constraints. Instead, the patient was initiated on triple IST consisting of ATG, cyclosporine (CsA), and prednisone. Treatment included rabbit Antithymocyte immunoglobulin (rATG) at 40 mg/kg/day intravenously for four consecutive days, in combination with Cyclosporine A at 5 mg/kg every 12 hours and methylprednisolone at 1 mg/kg every 12 hours^[6]^. The patient experienced no adverse effects or complications related to IST.

Despite this therapy, the patient showed no significant clinical response. Pancytopenia persisted (Table 1), and the patient continued to experience frequent hospital admissions. The patient required regular blood transfusions to manage ongoing anemia and thrombocytopenia.

The family is fully aware of the seriousness of their daughter’s condition and expressed deep distress over the fact that her life-saving treatment is limited by financial constraints. They reported feeling overwhelmed, as she remains on the waiting list for funding to undergo HSCT.

## Discussion

This case report describes a rare instance of AA following a URTI. She presented with normal vital signs and diffuse ecchymosis and purpura on her lower extremities. After investigation of the case, the patient was diagnosed with severe aplastic anemia (SAA).

It is a chronic primary hematopoietic failure that results in decreased hematopoietic precursors present in the bone marrow and attendant pancytopenia^[7]^. Two connected causes exist for AA: intrinsic abnormality of marrow progenitors and extrinsic immune-mediated suppression of hematopoietic stem cells^[8,9]^. Laboratory-wise, AA is defined as a decrease in blood counts with ≥2 hematopoietic lineages (i.e. absolute reticulocyte count <60 × 10^9^/L, absolute neutrophil count <0.5 × 10^9^/L, or platelet count <20 × 10^9^/L) and bone marrow hypocellularity (<25% of the normal cellularity)^[10]^.

To be able to kill other hematopoietic stem cells, damaged cells become self-reactive T helper cells (T1), which secrete cytokines, especially interferon alpha and tumor necrosis factor. Moreover, there is an upregulation in the apoptosis genes and death pathways^[11]^. The viruses more commonly associated with AA include hepatitis viruses, parvovirus, and HIV^[12]^. In our case, viral etiology was ruled out, and no apparent cause was evident after full investigation; thus, a diagnosis of idiopathic AA was made.

As for the treatment, although HSCT is the final treatment, this option is not always available. Instead, combining immunosuppressive medication with ATG and cyclosporine A will be considered the first-line treatment^[13]^. In our case, financial difficulties were the main barrier to bone marrow transplantation. That’s why the patient received ATG and cyclosporine A. These two drugs work by inhibiting T lymphocyte production and activity^[14,15]^. Horse ATG (hATG) is the recommended ATG source^[2]^, but in this case, rabbit ATG (rATG) was used instead; only rATG was accessible. The average time to respond with this regimen is reported to be approximately 3–4 months (range 1–13 months)^[16]^. In our case, we anticipated a response by around 3 months, which is consistent with published timelines for pediatric SAA treated with rabbit ATG.

A randomized controlled trial done with acquired AA showed that rATG is less effective compared to hATG^[17]^. Similar results were duplicated in the pediatric population where a multicentric analysis in 455 children showed higher infection rate and lower overall survival rate with rATG vs hATG^[18]^. Therefore, the European Working Group on Myelodysplastic Syndromes recommends hATG and considers the use of rATG acceptable if hATG is unavailable. In fact, lower day 180 response was noted with rATG (22%) compared to the hATG (42%)^[19]^. A retrospective analysis of 26 pediatric patients with AA confirmed poor response rate with rATG compared to hATG. This study underlines also the lack of resources and their effect on the prognosis, where in Brazil hATG is not present^[20]^.On the other hand, another comparative study done in Turkey on 15 pediatric patients showed partial responses after receiving rATG^[21]^.

Other second-line therapy exists for SAA, such as Thrombopoietin receptor agonist (TPO-RA). TPO-RA options for combination therapy with IST now include romiplostim in addition to eltrombopag, and studies have demonstrated the efficacy of triple therapy with ATG, CsA, and a TPO-RA^[22]^.

Studies indicate that for patients with SAA, HSCT is the most cost-effective option for those aged 18–35, while IST is preferred for patients aged 35–50 due to its balance of cost and quality-adjusted life years (QALYs). Although IST can induce remission, it is not curative and carries risks such as non-response, relapse, and clonal evolution. HSCT, while potentially curative, is more expensive and limited by donor availability and the risk of graft-versus-host disease^[23]^.

Literature has described varied outcomes of IST in patients with SAA who were unable to undergo HSCT. A 23-year-old woman with severe pancytopenia, ineligible for HSCT due to critically low leukocyte counts, received ATG, CsA, and eltrombopag but showed no hematologic improvement^[24]^. In contrast, a 35-year-old man who could not access allogeneic HSCT because of financial difficulties achieved complete trilineage recovery three months after initiating triple IST with ATG, CsA, and prednisone^[25]^. Similarly, a 25-year-old woman, in whom HSCT was deferred for financial reasons, demonstrated a markedly delayed hematologic response after 18 months of therapy, after receiving rabbit ATG and CsA^[26]^. These cases highlight both the heterogeneity of responses to IST and the potential for delayed, but meaningful, recovery in some patients with AA.

In resource-limited settings, financial constraints can delay access to definitive therapies. Consequently, physicians are often placed in a difficult position, balancing the urgency of initiating first-line therapies for life-threatening conditions against the feasibility of delivering such treatments. At present, the patient continues to receive supportive care, including regular blood and platelet transfusions, while awaiting the family’s ability to afford HSCT.

## Conclusion

This case highlights the complexities of diagnosing and managing severe AA in a pediatric 7-year-old girl with limited access to HSCT due to socioeconomic difficulties. Despite adhering to the standard guidelines of care and administering IST, the patient didn’t recover, emphasizing the variable responsiveness to IST and the critical need for alternative therapeutic strategies. Particularly in settings where HSCT is not accessible, physicians face challenges in managing refractory diseases with limited resources. This case report underscores the necessity for accessible and systemic healthcare solutions to ensure proper management and to bridge the equity gap in pediatric hematology and oncology care.

## Data Availability

Not applicable.

## References

[1] Segel GB Lichtman MA. Aplastic anemia: acquired and inherited. In: Kaushansky K Lichtman MA Prchal JT Levi MM Press OW Burns LJ eds. Williams Hematology, 9th ed. New York, NY: McGraw-Hill Education; 2015. accessmedicine.mhmedical.com/content.aspx?aid=1147670881

[2] Peslak SA Olson T Babushok DV. Diagnosis and Treatment of Aplastic Anemia. Curr Treat Options Oncol 2017;18:70. 29143887 10.1007/s11864-017-0511-z PMC5804354

[3] Young NS Calado RT Scheinberg P. Current concepts in the pathophysiology and treatment of aplastic anemia. Blood 2006;108:2509–19. 16778145 10.1182/blood-2006-03-010777 PMC1895575

[4] Killick SB Bown N Cavenagh J. Guidelines for the diagnosis and management of adult aplastic anaemia. Br J Haematol 2016;172:187–207. 26568159 10.1111/bjh.13853

[5] (PDF) Transparency in the reporting of Artificial INtelligence – the TITAN guideline. cited [31 Aug 2025]. https://www.researchgate.net/publication/392003420_Transparency_In_The_reporting_of_Artificial_INtelligence_-_the_TITAN_guideline

[6] Shimano KA Rothman JA Allen SW. Treatment of newly diagnosed severe aplastic anemia in children: evidence-based recommendations. Pediatr Blood Cancer 2024;71:e31070. 38757488 10.1002/pbc.31070

[7] Xue DS Chen T Wang T. The risk of clonal evolution of granulocyte colony-stimulating factor for acquired aplastic anemia: a systematic review and meta-analysis. Acta Haematol 2018;140:141–45. 30253387 10.1159/000491816

[8] Yamazaki H. Acquired aplastic anemia: recent advances in pathophysiology and treatment. Rinsho Ketsueki 2018;59:711–15. 29973449 10.11406/rinketsu.59.711

[9] Schoettler ML Nathan DG. The pathophysiology of acquired aplastic anemia: current concepts revisited. Hematol Oncol Clin North Am 2018;32:581–94. 30047412 10.1016/j.hoc.2018.03.001 PMC6538304

[10] DeZern AE Churpek JE. Approach to the diagnosis of aplastic anemia. Blood Adv 2021;5:2660–71. 34156438 10.1182/bloodadvances.2021004345 PMC8270669

[11] Nimmana BK Penney SW. Aplastic Anemia. In: StatPearls. Treasure Island (FL): StatPearls Publishing; 2025. http://www.ncbi.nlm.nih.gov/books/NBK534212/30480951

[12] Hinojosa OA Ammari O Hinojosa OA. Herpes simplex virus-associated aplastic anemia. Cureus 2023;15. 10.7759/cureus.35320 PMC10042545 36994301

[13] Piekarska A Pawelec K Szmigielska-Kapłon A. The state of the art in the treatment of severe aplastic anemia: immunotherapy and hematopoietic cell transplantation in children and adults. Front Immunol 2024;15:1378432. https://www.frontiersin.org/journals/immunology/articles/10.3389/fimmu.2024.1378432/full 38646536 10.3389/fimmu.2024.1378432 PMC11026616

[14] Tapia C Nessel TA Zito PM. Cyclosporine. In: StatPearls. Treasure Island, (FL): StatPearls Publishing; 2025. http://www.ncbi.nlm.nih.gov/books/NBK482450/

[15] Zhang L Jing L Zhou K. Rabbit antithymocyte globulin as first-line therapy for severe aplastic anemia. Exp Hematol 2015;43:286–94. 25583265 10.1016/j.exphem.2014.12.002

[16] Chen C Xue HM Xu HG. Rabbit-antithymocyte globulin combined with cyclosporin A as a first-line therapy: improved, effective, and safe for children with acquired severe aplastic anemia. J Cancer Res Clin Oncol 2012;138:1105–11. 22402596 10.1007/s00432-012-1184-4 PMC11824805

[17] Scheinberg P Nunez O Weinstein B. Horse versus rabbit antithymocyte globulin in acquired aplastic anemia. N Engl J Med 2011;365:430–38. 21812672 10.1056/NEJMoa1103975 PMC3721503

[18] Jeong DC Chung NG Cho B. Long-term outcome after immunosuppressive therapy with horse or rabbit antithymocyte globulin and cyclosporine for severe aplastic anemia in children. Haematologica 2014;99:664–71. 24213150 10.3324/haematol.2013.089268 PMC3971076

[19] Treatment | University Hospital Freiburg [cited 31 Aug 2025]. https://ewog-mds-saa.org/treatment.html

[20] Garanito MP Carneiro JDA Filho VO. Outcome of children with severe acquired aplastic anemia treated with rabbit antithymocyte globulin and cyclosporine A. J Pediatr Engl Ed 2014;90:523–27. 10.1016/j.jped.2014.02.004 24878006

[21] Karapinar DY Karadaş N Ay Y. Rabbit antithymocyte globulin treatment in childhood acquired severe aplastic anemia. Pediatr Hematol Oncol 2014;31:20–28. 23627541 10.3109/08880018.2013.792894

[22] Hosokawa K. A new era in the treatment of aplastic anemia. Rinsho Ketsueki 2025;66:572–79. 40769915 10.11406/rinketsu.66.572

[23] Zhang MX Wang Q Wang XQ. Hematopoietic stem-cell transplantation versus immunosuppressive therapy in patients with adult acquired severe aplastic anemia: a cost-effectiveness analysis. Int J Gen Med 2021;14:3529–37. 34290524 10.2147/IJGM.S310844 PMC8289465

[24] Bădulescu OV Popescu D Bădescu MC. Unresponsive severe aplastic anemia in a young patient: case report and short review of the literature. J Interdiscip Med 2021;6:116–19.

[25] Proskuriakova E Jasaraj RB San Hernandez AM. A case of severe aplastic anemia in a 35-year-old male with a good response to immunosuppressive therapy. Cureus 2023;15:e40210. 37435252 10.7759/cureus.40210 PMC10332331

[26] Alzahrani N Ashor N Fathi T. Idiopathic severe aplastic anemia with a delayed response to immunosuppressive therapy: a case report. Clin Case Rep 2018;6:1029–32. 29881557 10.1002/ccr3.1517 PMC5986045

